# Effects of different starter culture combinations on microbial counts and physico‐chemical properties in dry fermented mutton sausages

**DOI:** 10.1002/fsn3.989

**Published:** 2019-05-08

**Authors:** Debao Wang, Lihua Zhao, Rina Su, Ye Jin

**Affiliations:** ^1^ College of Food Science and Engineering Inner Mongolia Agricultural University Hohhot China; ^2^ Inner Mongolia Academy of Agriculture and Animal Husbandry 22 zhaojun road Hohhot China

**Keywords:** fermented mutton sausages, lipolysis, physico‐chemical properties, proteolysis, starter cultures

## Abstract

This study was conducted to evaluate the effects of inoculation with different mixed starter culture combinations on microbial counts, physico‐chemical properties, and proteolytic and lipolytic properties of dry fermented mutton sausages during fermentation and ripening. Four different batches of mutton sausages were manufactured: CO batch, no starter cultures, used as control; LB batch with *Lactobacillus sakei*; LS batch with *L. sakei* +* Staphylococcus xylosus*; and LSS batch with *L. sakei *+ *S. xylosus *+* Staphylococcus carnosus*. The results showed that adding starter culture caused *Lactobacillus* and *Staphylococcus* to become dominant bacteria and reduced the Enterobacteriaceae count in the inoculated sausages. The mixed starter cultures (LS & LSS) accelerated acidification and reduced water activity and lipid oxidation. Statistical analysis revealed that the use of mixed starter cultures, especially the combination *L. sakei* + *S. xylosus* + *S. carnosus*, contributed to proteolysis and lipolysis, increasing total free amino acids and polyunsaturated fatty acids. The above results demonstrate that the use of mixed starter cultures contributes to improving the composition of free amino acids and free fatty acids as well as the hygienic quality of dry fermented mutton sausages.

## 1 INTRODUCTION

Fermented sausages are produced under certain temperature and relative humidity conditions by the process of microbiological, biochemical, and physical processes and sensory property change during ripening (Casaburi et al., 2007). However, the quality and safety of sausages fermented by traditional means of spontaneous fermentation may not be guaranteed. Due to changes in shipping and consuming habits, the use of starter cultures in dry fermented sausages has become increasingly useful for ensuring safety and extending shelf life by reducing pH and water activity (a_w_) and inhibiting the development of pathogenic and spoilage bacteria (Ciuciu Simion, Vizireanu, Alexe, Franco, & Carballo, 2014; Essid & Hassouna, 2013). During fermentation and ripening of dry fermented sausages, meat proteins are degraded to oligopeptides and free amino acids produced by histenzyme and aminopeptidase, which are partly secreted by starter cultures. The products of protein degradation contribute significantly to the flavor of the sausages (Stahnke & Marie, 1999). Commonly used microbial starter cultures include lactic acid bacteria (LAB) and some staphylococcal species, such as *Lactobacillus sakei*,* Staphylococcus xylosus,* and *Staphylococcus carnosus*, as commercial starters for the manufacture of several types of dry fermented sausages (Corbière Morot‐Bizot, Leroy, & Talon, 2006). Staphylococci and lactic acid bacteria can help methyl branched amino acids synthesize into 2‐ and 3‐methyl butanal thus improving the flavor of dried‐fermented sausage (Schmidt & Berger, 1998; Stahnke & Marie, 1999). In addition, hexanal, octanal, and nonanal caused by β‐oxidation of lipids are related not only to substrate concentration but also to microbial starter activity (Olivares, Navarro, & Flores, 2011). Mutton is characterized by high protein, low fat, and low cholesterol content, and using mutton as raw meat for dry fermented sausages can increase the nutritional value of fermented sausages. To our knowledge, studies on different mixed starter cultures inoculated in dry fermented mutton sausages during fermentation and ripening are rare. The aim of this work was to evaluate the effect of different mixed starter culture combinations on the microbiological and physico‐chemical properties and free amino acid and free fatty acid composition of dry fermented mutton sausages.

## 2 MATERIALS AND METHODS

### 2.1 Dry fermented sausage preparation and sampling procedures

The sausages were low temperature fermentation mutton sausages manufactured in the meat production center of Inner Mongolia Agricultural University of China, while the Food Microbiology Lab of the center provided the starter cultures of *L. sakei*,* S. xylosus,* and *S. carnosus*. Dry fermentation mutton sausage formulation includes lean mutton meat (80% w/w), tallow (20% w/w), sucrose (0.5% w/w), glucose (0.5% w/w), ascorbic acid (0.05% w/w), sodium nitrate (0.015% w/w), and nitrite (0.01% w/w). Four different batches of mutton sausages were included as follows: (a) CO batch, without commercial starter cultures; (b) LB batch, with *L. sakei*; (c) LS batch, with *L. sakei* +* S. xylosus*; and (d) LSS batch, with mixed starter cultures of *L. sakei* + *S. xylosus *+ *S. carnosus*. Mutton meat and fat were made into 4–6 mm particle sizes, which were mixed with starter cultures and other ingredients. The mixture was pickled for 24 hr at 0–4°C and then stuffed into collagen casings with a diameter of 30 mm and length of 15 cm. First, the sausages were fermented for 2 days at 25°C and 90%–95% relative humidity (RH). After fermentation, the sausages entered the dry‐ripening process for 10 days at 14–15°C and 75%–85% (RH). Samples were obtained at 0 (end of pickling), 2, 5, and 12 days for subsequent experimental analyses.

### 2.2 Microbial analysis

Microbiological analyses were performed at the end of the pickling, fermentation, drying, and ripening processes using the methods of Wang et al. (2015) with a slight modification. After aseptically removing and discarding the outer casing, 10 g of each sample was weighed in a sterile plastic bag. Then, samples were homogenized with 90 ml of sterile physiological saline at 20–25°C for 2 min in a stomacher (Seward Medical, London, UK) and were 10‐fold diluted in sterile 0.1% peptone water and 0.85% NaCl. Total viable counts were analyzed using plate count agar (PCA) after incubation at 30°C for 48 hr. Lactic acid bacteria and staphylococci were enhanced using MRS agar and MSA agar after incubation at 30°C for 48 hr. Enterobacteriaceae were determined on VRBG Agar after incubation at 30°C for 48 hr.

### 2.3 pH, water activity, and thiobarbituric acid‐reactive substances (TBARS) analysis

The sausage pH and water activity were measured using a digital pH meter (Mettler Toledo, Shanghai, China) and LabMaster‐aw (Novasina AG, Switzerland). Ten × 10 mm samples (diameter × height) were compressed at a probe (P/36R) speed of 1 mm/s using a texture analyzer (TA.XT2i, SMS, Germany). Texture analysis was performed by compressing 50% using a P/36R probe, and each sample was compressed twice and the interval time was 5 s. The effect of starters on the oxidative stability of fermented mutton sausages was evaluated by measuring TBARS values according to the methods of Bingol et al. (2014) with a slight modification. Sausage samples (0.2 g) were taken, and TBARS values were extracted for 1 hr at 4°C with 4.25 ml of TBARS solution containing 0.28% TBARS, 0.009% BHT, 0.4% SDS, 1.2 M acetate buffer, and pH 3.5, and the extract was heated in a boiling water bath at 90°C for 60 min. After cooling, 1 ml of distilled water and 5 ml of n‐butyl alcohol:pyridine (15:1) were added to the extracts and mixed using a vortex mixer. The mixtures were centrifuged at 1509.3 × *g* for 10 min at room temperature. After centrifugation, the upper phase was pipetted into test tubes. Sample absorbance was read against the appropriate blank at 532 nm. TBARS was expressed as mg of malondialdehyde (MDA) per kilogram of meat. The formula is as follows:

TBARS value = ([absorbance‐0.0121]/0.1379) × (72.06/94) mg MDA/kg sausage.

### 2.4 Proteolysis index (PI) analysis

Proteolysis index of sausages was determined by the methods of Hughes et al. (2002). Two grams of test samples was diluted in 18 ml distilled water. The solution was homogenized for 2 min and centrifuged at 1,000 *g* at 4°C for 15 min (AllegraTM, 64R, Beckman, American). The above supernatant was filtered using Whatman #1 filters (Mosutech, Shanghai, China). The extraction process was repeated once, and the merged filtrate was subjected to the tests. Fifteen milliliters of test fluid described above was mixed with 15 ml 10% trichloroacetic acid and then filtered by Whatman #1 filters. Five milliliters of the resulting filtrate was measured for nonprotein nitrogen content using Kjeldahl nitrogen determination instruments (Jinghe analytical instruments, Shanghai, China). Protein content of the sausage was determined by the same instrument. The formula for the protein decomposition index is as follows:

PI = 0.2 V × N_1_/N;

where V: filtrate; N_1_: nonprotein nitrogen content; and N: protein content in the sausage.

### 2.5 Free amino acid analysis

Free amino acid content was analyzed by high‐performance liquid chromatography (HPLC) according to the per‐column derivatization method. The samples were dried at temperatures < 63°C to a constant weight, and the fat of the dried samples was removed with ether (Sinopharm Chemical Reagent Co., Shanghai, China). Thirty milligrams of sample was crushed and added to a long neck tube and extracted using 0.1M HCl. Then, vacuum samples were hydrolyzed at 100°C for 24 hr and filtered and fixed to a 50‐ml volumetric flask. In addition, 150 μl of the mixture described above was mixed with 50 μl 60 mM adjacent nitrobenzene sulfonic acid chloride and 1 ml 0.05 mM borax buffer solution (adjusted to pH 9.0 with acetic acid), which were derived at 25°C for 10 min. The above were filtered using a 0.45‐μm filter for high‐performance liquid chromatography analysis. The free amino acid results were expressed in mg/kg of dry matter.

### 2.6 Free fatty acid analysis

Total lipids were extracted according to the method of Folch, Lees, and Sloane Stanley (1957) with a slight modification using chloroform: methanol (2:1) as the extracted solvent. The extracts were concentrated in a rotating vacuum evaporator. Free fatty acids were determined as described by Olivares et al. (2011) and expressed in mg/100 g of fat.

### 2.7 Sensory analysis

The sausages were submitted to sensory evaluation to determine the effect of processing method and starter inoculation on the quality of final product. Appearance, color, flavor, texture, and overall quality attributes were evaluated using a hedonic scale with nine points (1 = very bad, 9 = very good). The sensory panel consisted of nine trained panelists. Tests were conducted at 20–22°C in a well‐ventilated room. Samples were sliced to 4 mm thickness and held in a 5‐mm‐diameter plastic container with cover. Water and bread were provided for panelists to rinse and clean their mouths between samples. Sensory data were analyzed using the nonparametric Mann–Whitney test to determine the effect of processing method and starter inoculation (Gibbons, 1976).

### 2.8 Statistical analysis

All statistical analyses were performed using IBM SPSS Statistics 19 software (IBM, Chicago, IL, USA). The statistical significance (*p *<* *0.05) was determined using one‐way ANOVA. Duncan's test and the LSD method were performed to compare the mean values during processing time.

## 3 RESULTS AND DISCUSSION

### 3.1 Effect of starter culture combinations on microbial counts during fermentation and ripening

The effect of different starter cultures on microbial counts of dry fermented mutton sausages is shown in Table 1. Statistical analysis showed a significant difference (*p *<* *0.01) between the control and the inoculated microbial counts. This result was similar to Essid and Hassouna (2013), who reported that using the selective starter significantly influenced the total viable counts of PCA, staphylococci, LAB, and Enterobacteriaceae. LAB counts in all batches were very close to the total viable number. Initial LAB counts in LB, LS, and LSS were three log units higher than the control (6.74 vs. 6.77 vs. 6.74 vs 3.69 log CFU/g, respectively). The LAB counts in the four groups increased gradually until reaching maximum levels on the 5th day of ripening, and a slight decrease was observed at the end of ripening, which could be due to the decrease in carbohydrate content and decline of water activity in fermented sausages (Lorenzo & Franco, 2012). In addition, LAB were the most competitive microorganisms, which may be due to LAB being well adapted to the meat environment and to a positive interaction among species (Essid & Hassouna, 2013; Lorenzo & Franco, 2012; Zhao et al., 2011). LAB contribute to the development of the physico‐chemical qualities of fermented sausages, such as texture, flavor, hygiene, and safety‐related properties (Essid & Hassouna, 2013). During the whole process of fermentation and ripening, LAB and staphylococci counts were significantly higher in LB, LS, LSS than in the control (*p *<* *0.001). As reported by Lu et al. (2010), staphylococci were the second predominant bacteria in mixed starter batches, and the growth rate of the inoculated batches was faster than that of the control. At day 5, staphylococci counts reached maximum levels (8.14, 9.08, and 9.10 log CFU/g for LB, LS, and LSS batches, respectively). However, the staphylococci count in the inoculated batches decreased by approximately 7.42%–8.88% at the end of ripening. This could be due to the poor competitiveness of staphylococci and the decrease in pH and water activity in the sausage, as reported in other works (Essid & Hassouna, 2013; Zhao et al., 2011). During fermentation and ripening, Enterobacteriaceae counts decreased gradually in the four treatment groups, and the inoculated batches had significantly lower Enterobacteriaceae counts than those of the control (4.90, 3.93, 3.73, and 3.94 log CFU/g for CO, LB, LS, and LSS batches) on day 5 (*p *<* *0.01), which may be the result of the lower a_w_ and pH values in the sausage and growth of LAB (Lorenzo, Gomez, & Fonseca, 2014; Lorenzo, Sarriés et al., 2014). At day 12, Enterobacteriaceae counts of LB, LS, and LSS batches (2.36, 2.80, and 2.38 log CFU/g, respectively) presented significantly (*p *<* *0.001) lower than those of the control (3.41 log CFU/g), which agreed with the report of Tabanelli et al. (2012). A lower amount of Enterobacteriaceae improves the product quality and safety of dry fermented sausages by producing less harmful substances, such as biogenic amines (Lu et al., 2010; Ma et al., 2015).

**Table 1:** Effect of different mixed starter cultures on microbial counts (log CFU/g) of total mesophilic aerobic bacteria (PCA), staphylococci (MSA), lactic acid bacteria (MRS), and Enterobacteriaceae (VRBGA) of dry fermented sausages at various processing stage (means ± *SD* of six replicates)

Microbiological counts	Days	Batch				Significance
		CO	LB	LS	LSS	
Total viable counts	0	5.87 ± 0.11^Aa^	6.49 ± 0.12^Ab^	6.42 ± 0.03^Ab^	6.86 ± 0.02^Ac^	**
	2	9.13 ± 0.06^Ba^	9.25 ± 0.05^Ba^	9.17 ± 0.05^Ba^	9.66 ± 0.01^Bb^	**
	5	9.32 ± 0.13^Ba^	9.73 ± 0.09^Bb^	9.64 ± 0.11^Bab^	9.50 ± 0.10^Bab^	**
	12	8.20 ± 0.08^Ca^	8.38 ± 0.07^Cb^	8.87 ± 0.33^Cc^	8.82 ± 0.03^Cc^	**
	Significance	***	***	***	***	
Lactic acid bacterial	0	3.69 ± 0.06^Aa^	6.74 ± 0.13^Ab^	6.77 ± 0.25^Ab^	6.74 ± 0.03^Ab^	***
	2	7.52 ± 0.23^Bab^	8.48 ± 0.01^Bb^	8.50 ± 0.12^Bb^	8.55 ± 0.21^Bb^	**
	5	7.92 ± 0.03^Ba^	9.28 ± 0.32^Cb^	9.24 ± 0.26^Cb^	9.61 ± 0.12^Dc^	***
	12	7.39 ± 0.18^Ba^	9.18 ± 0.02^Cb^	9.18 ± 0.03^Cb^	9.13 ± 0.26^Cb^	**
	Significance	***	***	***	***	
Staphylococci	0	4.06 ± 0.03^Aa^	5.30 ± 0.05^Ab^	6.13 ± 0.05^Ac^	6.36 ± 0.11^Ac^	***
	2	7.83 ± 0.02^Ba^	7.92 ± 0.07^B,a^	8.84 ± 0.06^Bb^	9.06 ± 0.02^Bb^	**
	5	7.70 ± 0.06^Ba^	8.14 ± 0.03^Ba^	9.08 ± 0.03^Bb^	9.10 ± 0.02^Bb^	***
	12	7.19 ± 0.13^Ca^	7.45 ± 0.15^Ba^	8.28 ± 0.17^Cb^	8.45 ± 0.01^Cb^	***
	Significance	***	***	***	***	
Enterobacteriaceae	0	4.13 ± 0.19^Ba^	3.98 ± 0.02^Ba^	4.02 ± 0.04^Ba^	3.92 ± 0.04^Ba^	n.s.
	2	5.72 ± 0.02^Cb^	4.78 ± 0.03^Ca^	5.31 ± 0.16^Cb^	4.55 ± 0.04^Ca^	***
	5	4.90 ± 0.04^Bc^	3.93 ± 0.02^Bb^	3.73 ± 0.08^Ba^	3.94 ± 0.01^Bb^	**
	12	3.41 ± 0.07^Ac^	2.36 ± 0.03^Aa^	2.80 ± 0.01^Ab^	2.38 ± 0.02^Aa^	***
	Significance	***	***	***	***	

Notes

Batches: CO: without commercial starter cultures; LB: with single Lactobacillus starter culture (*L. sake*); LS: with compound starter cultures (*L. sake* + *S. xylosus*); LSS: with compound starter cultures (*L. sake* + *S. xylosus* + *S. carnosus*).

^a,b,c^Mean values in the same row (corresponding to the same days of ripening) not followed by a common number differ significantly (*p* < 0.05).

^A,B,C,D^Mean values in the same row (corresponding to the same batch) not followed by a common letter differ significantly (*p *<* *0.05).

Significance: n.s.: not significant; *(*p *<* *0.05); **(*p *<* *0.01); ***(*p *<* *0.001).

### 3.2 Effect of starter cultures on pH, water activity, TBARS, and protein decomposition index of dry fermented mutton sausages

The changes in pH, water activity, TBARS, and the protein decomposition index of dry fermented mutton sausages during fermentation and ripening are presented in Table 2. The initial pH values of 5.46, 5.49, 5.45, and 5.41 for the control, LB, LS, and LSS batches decreased sharply (*p *<* *0.01) to 5.25, 5.02, 4.85, and 4.86 on day 2, respectively. The inoculated batches reached lower values (4.55, 4.50, and 4.40) compared with the control (4.89) at day 5. This indicated that the inoculation of starter cultures prompted carbohydrate breakdown and the accumulation of organic acids, mainly lactic acids, which led to a significantly faster acidification rate of the inoculated batches than that of the control, which agreed with the report by Nie, Lin, and Zhang (2014) and Zhao et al. (2011). The rapid decline of pH value during fermentation is critical because it helps to inhibit the growth of undesirable microorganisms and improve fermented sausages with redder color (Lorenzo, Gomez, et al., 2014; Lorenzo, Sarriés, et al., 2014). The increase in pH values in the inoculated sausages at 12 days may be caused by the increase in proteolytic peptides and amines, which was induced by bacterial proteases (Ruiz‐Moyano et al., 2011). It is essential to ensure the drying process of mutton sausages by stronger acidification to reduce the water‐binding capacity of proteins and promote water evaporation (Lorenzo, Gomez, et al., 2014; Lorenzo, Sarriés, et al., 2014). At the beginning of the production of the fermented sausages, the water activity (a_w_) of all four batches was above 0.95. After 2 days, the a_w_ of the inoculated batches decreased significantly (*p *<* *0.001) compared with the control, and all batches decreased to the lowest a_w_ at the end of ripening. This result disagreed with the study of Lorenzo, Gomez, et al. (2014) and Lorenzo, Sarriés, et al. (2014) and was similar to Kaban and Kaya (2009). Lower a_w_ at the end of ripening improves fermented sausage quality and extends shelf life. According to the report of Ulu (2004), the TBARS value was used to evaluate the lipid oxidation level of fermented sausages. TBARS values ranging from 0.6 to 2.8 mg MDA/kg are considered to be normal in fermented sausages (Marco, Navarro, & Flores, 2006). In this study, TBARS values presented an increasing trend, from an initial value of 0.35 mg MDA/kg reached to 0.62, 0.53, 0.52, and 0.49 at the end of ripening for the CO, LB, LS, and LSS batches, respectively. However, the TBARS values of the inoculated batches were lower (*p *<* *0.001) compared with the CO in the fermentation and ripening period. Sun, Chen, Li, Zheng, and Kong (2016) demonstrated that LAB and staphylococcus had higher antioxidant activity and inhibition of lipid oxidation than other strains in vitro. The present study showed that the inoculated starters exhibited potential antioxidant ability may be due to their antioxidant enzyme system (such as superoxide dismutase, catalase, and glutathione peroxidase) suppressing substances (such as uric acid, glutathione, polysaccharides, NADH, and NADPH) (Lin & Yen, 1999). Proteolysis plays an important role in biochemical changes during fermentation and ripening of fermented sausages and improves sausage quality with regard to color, texture, and flavor (Benito, Rodríguez, Córdoba, Andrade, & Córdoba, 2005). At the beginning of the process, the proteolysis indexes of the control, LB, LS, and LSS batches were 26.47%, 26.47%, 26.47%, and 26.43%, respectively, which showed no significant differences (*p *>* *0.05). Between days 2 and 5, the increasing amplitude of the proteolysis index of all batches was the largest, but the proteolysis index of the inoculated batches was significantly higher than that of the CO batches (*p *<* *0.001). All inoculated starter cultures tested possessed a certain degree of protein hydrolysis activity. Similar results were observed with silver carp sausages (Xu, Xia, Yang, & Nie, 2010). The research of Nie et al. (2014) by SDS‐PAGE profiles suggested that LAB could promote the degradation of myofibrillar and sarcoplasmic proteins.

**Table 2:** Effect of different mixed starter cultures on pH, Aw, TBARS, and PI of dry fermented mutton sausage at various processing stages (means ± *SD* of six replicates)

Days		Batch				Significance
		CO	LB	LS	LSS	
pH	0	5.46 ± 0.01^Db^	5.49 ± 0.12^Dc^	5.45 ± 0.05^Cb^	5.41 ± 0.00^Da^	***
	2	5.25 ± 0.02^Cc^	5.02 ± 0.02^Cb^	4.85 ± 0.01^Ba^	4.86 ± 0.05^Ca^	***
	5	4.89 ± 0.00^Bc^	4.55 ± 0.05^Ab^	4.50 ± 0.03^Ab^	4.40 ± 0.04^Aa^	***
	12	4.68 ± 0.04^Ac^	4.77 ± 0.02^Bd^	4.50 ± 0.04^Aa^	4.60 ± 0.00^Bb^	***
	Significance	***	***	***	***	
a_w_	0	0.954 ± 0.002^Ba^	0.958 ± 0.002^Ca^	0.956 ± 0.002^Ca^	0.953 ± 0.005^Ca^	n.s.
	2	0.947 ± 0.001^Ba^	0.947 ± 0.001^Ca^	0.947 ± 0.002^Ca^	0.946 ± 0.003^Ca^	n.s.
	5	0.932 ± 0.005^Bd^	0.892 ± 0.003^Bc^	0.860 ± 0.00^Bb^	0.834 ± 0.005^Ba^	*
	12	0.714 ± 0.007^Abc^	0.724 ± 0.012^Ac^	0.686 ± 0.007^Aab^	0.666 ± 0.009^Aa^	*
	Significance	***	***	***	***	
TBARS	0	0.35 ± 0.00^Aa^	0.35 ± 0.01^Aa^	0.36 ± 0.01^Aa^	0.35 ± 0.00^Aa^	n.s.
	2	0.47 ± 0.01^Bb^	0.40 ± 0.02^Ba^	0.41 ± 0.01^Ba^	0.39 ± 0.00^Ba^	***
	5	0.52 ± 0.06^Cc^	0.44 ± 0.20^Cb^	0.41 ± 0.04^Ba^	0.42 ± 0.06^Ca^	***
	12	0.62 ± 0.12^Dc^	0.53 ± 0.05^Db^	0.52 ± 0.03^Cc^	0.49 ± 0.00 ^Da^	***
	Significance	***	***	***	***	
PI (%)	0	26.47 ± 2.31^Aa^	26.47 ± 1.55^Aa^	26.47 ± 0.40^Aa^	26.43 ± 1.53^Aa^	n.s.
	2	27.50 ± 2.96^Bb^	27.11 ± 2.85^Ba^	28.37 ± 0.70^Bc^	27.50 ± 2.59^Bb^	***
	5	27.80%±2.38^Ca^	28.13 ± 4.39^Cb^	30.67 ± 1.99^Cd^	29.93 ± 3.06^Cc^	***
	12	29.00 ± 1.50^Da^	29.83 ± 0.25 ^Da^	31.00 ± 1.69^Dc^	30.07 ± 1.15^Db^	***
	Significance	***	***	***	***	

Notes

Aw, water activity; pH, pH; PI, protein decomposing index; TBARS, thiobarbituric acid‐reactive substances.

Batches: CO: without commercial starter cultures; LB: with single Lactobacillus starter culture (*L. sake*); LS: with compound starter cultures (*L. sake* + *S. xylosus*); LSS: with compound starter cultures (*L. sake* + *S. xylosus* + *S. carnosus*).

^a,b,c,d^Mean values in the same row (corresponding to the same days of ripening) not followed by a common number differ significantly (*p* < 0.05).

^A,B,C,D^Mean values in the same row (corresponding to the same batch) not followed by a common letter differ significantly (*p* < 0.05).

Significance: n.s.: not significant; *(*p *<* *0.05); **(*p *<* *0.01); ***(*p *<* *0.001).

### 3.3 Effect of starter cultures on free amino acids at the end of ripening

Free amino acids, as precursors of many odorants, participate indirectly in flavor development and contribute directly to the taste of sausage products. The analysis of free amino acids (FAA) (expressed as mg/kg of dry sausage samples) of dry fermented mutton sausage is reported in Table 3. The total FAA in LB, LS, and LSS batches was higher than in the control (*p *<* *0.001), which implied that the total FAA could be increased by inoculated starter cultures. However, the total FAA content did not differ significantly among the inoculated batches, which agree with the research of Casaburi et al. (2007) and Candogan, Wardlaw, and Acton (2009). It is well known that the release of free amino acids is attributable to the proteolytic action of microbial enzymes and endogenous enzymes in fermented sausages (Candogan et al., 2009). The types of amino acids play an important role in flavor and taste development (Bermúdez, Franco, Carballo, Sentandreu, & Lorenzo, 2014). As Mau & Tseng (1998) reported, glutamic acid and aspartic acid are the main amine acids that ‘impart fresh taste to food, glycine and alanine impart a sweet taste to food, while arginine, leucine, lysine, valine, and phenylalanine cause food to have a bitter taste. Other FAAs show sour or salty characteristics (Mau & Tseng, 1998). Therefore, the release and contents of FAA effect sausage flavor and taste. For example, sausages inoculated by *S. xylosus* and *S. carnosus* can produce 3‐methyl butyl aldehyde, which are important compositions of the dry, pickled flavor of fermented meat, by decomposing leucine, isoleucine, valine, and phenylalanine (Stahnke & Marie, 1999); 3‐methyl butyl aldehyde effects sausages by causing a bacon‐like flavor when it reacts with sulfur compounds (Hinrichsen & Andersen, 1994). Some FAA appeared to be the predominant amino acids in all of the final mixes, such as glutamate (10.38, 12.36, 12.05, and 13.09 mg/kg dry sausage for CO, LB, LS, and LSS batches, respectively), aspartic (5.82, 7.15, 6.94, and 7.42 mg/kg dry sausage for CO, LB, LS, and LSS batches), leucine (5.53, 6.55, 6.55, and 7.20 mg/kg dry sausage for CO, LB, LS, and LSS batches), and lysine (5.63, 6.82, 6.79, and 7.18 mg/kg dry sausage for CO, LB, LS, and LSS batches). This result suggested that *L. sakei* and *S. carnosus* were endowed with proteolytic activity, which played a main role in the release of free amino acids that affect flavor development. Some authors (Casaburi et al., 2007; Hughes et al., 2002) have reported that sarcoplasmic proteins are degraded by endogenous muscle and microbiological enzymes, indicating that exogenous proteases produced by starter culture and endogenous proteins play a significant role in proteolysis during the ripening of fermented sausages.

**Table 3:** Effect of different mixed starter cultures on free amino acids (expressed as mg/kg of dry matter) of dry fermented mutton sausage at the end of ripening (means ± *SD* of six replicates)

FAA	Batch				Significance
	CO	LB	LS	LSS	
Aspartic acid	5.82 ± 0.01^a^	7.15 ± 0.59^b^	6.94 ± 0.00^ab^	7.42 ± 0.05^b^	***
Threonine	2.91 ± 0.05^a^	3.62 ± 0.03^b^	3.51 ± 0.02^b^	3.61 ± 0.03^b^	***
Serine	2.29 ± 0.04^a^	2.73 ± 0.03^b^	2.78 ± 0.04^b^	2.78 ± 0.03^b^	***
Glutamic acid	10.38 ± 0.04^a^	12.36 ± 0.14^b^	12.05 ± 0.11^b^	13.09 ± 0.06^c^	***
Glycine	2.98 ± 0.07^a^	3.97 ± 0.01^c^	3.33 ± 0.09^b^	3.68 ± 0.09^c^	***
Alanine	3.78 ± 0.02^a^	4.78 ± 0.03^c^	4.36 ± 0.05^b^	4.72 ± 0.01^c^	***
Cystine	0.72 ± 0.00^a^	0.80 ± 0.01^b^	0.84 ± 0.01^c^	0. 80 ± 0.00^b^	***
Valine	3.22 ± 0.07^a^	3.76 ± 0.04^b^	3.72 ± 0.05^b^	4.04 ± 0.03^c^	***
Methionine	1.61 ± 0.01^a^	1.94 ± 0.01^b^	1.93 ± 0.12^b^	2.04 ± 0.01^b^	***
Isoleucine	3.05 ± 0.05^a^	3.50 ± 0.07^b^	3.55 ± 0.08^b^	3.97 ± 0.05^c^	***
Leucine	5.53 ± 0.01^a^	6.55 ± 0.19^b^	6.55 ± 0.09^b^	7.20 ± 0.10^c^	***
Tyrosine	1.73 ± 0.05^a^	2.45 ± 0.01^a^	2.25 ± 0.08^a^	2.27 ± 0.01^a^	n.s.
Phenylalanine	2.64 ± 0.07^a^	3.08 ± 0.04^b^	3.10 ± 0.08^b^	3.35 ± 0.02^c^	***
Lysine	5.63 ± 0.13^a^	6.82 ± 0.07^b^	6.79 ± 0.64^b^	7.18 ± 0.14^b^	***
Histidine	1.83 ± 0.00^a^	2.26 ± 0.01^b^	2.33 ± 0.07^c^	2.39 ± 0.01^c^	***
Arginine	4.17 ± 0.03^a^	5.10 ± 0.24^b^	4.97 ± 0.34^b^	5.33 ± 0.05^b^	***
Proline	1.52 ± 0.02^a^	3.10 ± 0.06^d^	1.77 ± 0.04^b^	1.88 ± 0.00^c^	***
Total free amino acids	59.81 ± 0.02^a^	73.97 ± 0.12^c^	70.69 ± 0.04^b^	75.75 ± 0.02^c^	***

Notes

Batches: CO: without commercial starter cultures; LB: with single Lactobacillus starter culture (*L. sake*); LS: with compound starter cultures (*L. sake* + *S. xylosus*); LSS: with compound starter cultures (*L. sake* + *S. xylosus* + *S. carnosus*).

^a,b,c,d^Mean values in the same row (corresponding to the same days of ripening) not followed by a common number differ significantly (*p *<* *0.05).

Significance: n.s.: not significant; *(*p *<* *0.05); **(*p *<* *0.01); ***(*p *<* *0.001).

### 3.4 Effect of starter cultures on free fatty acids during fermentation and ripening

The fatty acid compositions in dry fermented mutton sausages are shown in Table 4. The composition and content of fatty acids contribute to the nutritional value of fermented sausage. Appropriate ratio of omega‐6/omega‐3 fatty acid intake can improve dietary energy homeostasis, thus preventing humans from cardiovascular, stroke, and other diseases (Regulaka‐llow et al., 2013). In addition, lipolysis is directly involved in flavor formation during ripening of cured products (Casaburi et al., 2007). In this study, monounsaturated fatty acids (MUFA) were found at the highest levels followed by saturated fatty acids (SFA) and polyunsaturated fatty acids (PUFA) in the production process, which was similar to that reported by Alicia, José, and Mónica (2015). The total FFA in all batches showed a significant (*p *<* *0.05) increasing tendency from day 0 to day 5, and the total content changed from 599.30 to 899.17 mg/100 g of fat and had a small decrease from day 5 to day 12. However, the total FFA amounts in this study were lower than the research of Lorenzo, Gomez, et al. (2014), Lorenzo, Sarriés, et al. (2014) and Lorenzo, Fonseca, Gómez, and Domínguez (2015) in foal sausages and Trani et al. (2010) in pork dry‐cured sausages. The different contents of FFA among these studies were mainly due to the various raw materials, formulas, conditions of the processes, and distinct activity and specificity of the lipase of endogenous enzymes and starter cultures (Lorenzo & Franco, 2012). The FFA contents in the four groups showed the same tendency, increasing first and then decreasing during the process. The MUFA content (between 44% and 56% of total FFA) was significantly (*p *<* *0.05) higher than that of SFA (between 34% and 42% of total FFA), while PUFA (between 10% and 15% of total FFA) in the total FFA was the lowest. This result agreed with the reports of Rubén, Paulo, Rubén, and José (2016). Stearic acid (C18:0), as one of SFA, presented the highest level, followed by myristic acid (C14:0), heptadecanoic acid (C17:0), ginkgolic acid (C15:0), and palmitic acid (C16:0), and the sums of these five fatty acids were 92%–97% of the total SFA in the four groups. During the entire process, the change in myristic acid was the greatest. Oleic acid (C18:1) content was the highest in all MUFA, followed by palmitic acid (C16:1) and ginkgo acid (C17:1), whose content represented between 96% and 99% of the MUFA. Mottram (1998) showed that palmitic acid (C16:1) content has a significant positive correlation with meat flavor. Some PUFA appeared to be the predominant fatty acids, such as linoleic acid (C18:2 n6), linolenic acid (C18:3 n3), and eicosapentaenoic acid (C20:5 n3). Arachidonic acid (C20:4 n6) content was lower than other PUFA, which could be transformed into flavor substances with mushroom flavor by fat oxidation. Alpha‐linolenic acid (C18:3 n3) in the fermented dry mutton sausages accounted for percentages between 2% and 5% of the total FFA in all groups.

**Table 4:** Effect of different mixed starter cultures on free fatty acid (expressed as mg/100 g of fat) of dry fermented mutton sausage at various processing stage (means ± *SD* of six replicates)

FFA	Batch	Days of processing				Significance
		0	1	4	7	
C10:0	CO	2.69 ± 0.00^Cb^	2.36 ± 0.00^Aa^	4.53 ± 0.01^Cd^	3.14 ± 0.03^Ac^	***
	LB	2.16 ± 0.01^Aa^	2.72 ± 0.01^ABb^	4.50 ± 0.01^Cc^	4.27 ± 0.03^Bc^	***
	LS	2.23 ± 0.01^Ba^	2.88 ± 0.62^ABa^	3.99 ± 0.07^Ab^	4.88 ± 0.66^Bc^	**
	LSS	2.23 ± 0.01^Ba^	2.76 ± 0.00^Cb^	4.10 ± 0.02^Bd^	3.62 ± 0.06^Ac^	***
	Significance	***	*	***	*	
C12:0	CO	1.81 ± 0.00^Ba^	2.44 ± 0.00^Ab^	4.67 ± 0.01^Dd^	3.16 ± 0.01^Ac^	***
	LB	1.20 ± 0.01^Aa^	2.64 ± 0.01^Bb^	4.26 ± 0.01^Cd^	4.33 ± 0.02^Bc^	***
	LS	2.06 ± 0.00 ^Da^	3.50 ± 0.06^Cb^	3.55 ± 0.06^Ab^	4.29 ± 0.03^Bc^	***
	LSS	1.98 ± 0.00^Ca^	2.58 ± 0.00^Ab^	3.68 ± 0.07^Bd^	3.21 ± 0.05^Ac^	***
	Significance	***	***	***	***	
C14:0	CO	34.99 ± 0.00^Ba^	57.01 ± 0.03^Bb^	87.70 ± 0.16^Bc^	57.21 ± 0.16^Ab^	***
	LB	25.53 ± 0.15^Aa^	57.20 ± 0.19^Bb^	95.22 ± 0.09^Cd^	80.23 ± 0.00^Bc^	***
	LS	43.51 ± 0.06 ^Da^	52.39 ± 0.62^Aa^	74.87 ± 1.31^Ab^	80.40 ± 0.06^Bb^	*
	LSS	39.34 ± 0.12^Ca^	52.73 ± 0.14^Ab^	74.02 ± 0.34^Ad^	58.34 ± 0.15^Ac^	***
	Significance	***	***	***	***	
C15:0	CO	4.10 ± 0.02^Aa^	17.43 ± 0.12^Cc^	23.70 ± 0.07^Bd^	14.14 ± 0.04^Ab^	***
	LB	6.13 ± 0.03^Ba^	15.48 ± 0.02^Bb^	23.83 ± 0.09^Bc^	20.54 ± 0.11^Cc^	***
	LS	11.20 ± 0.03 ^Da^	14.71 ± 0.63^Bb^	18.83 ± 0.28^Ac^	21.26 ± 0.42^Dd^	***
	LSS	10.27 ± 0.04^Ca^	13.50 ± 0.01^Ab^	19.07 ± 0.59^Ad^	15.75 ± 0.01^Bc^	***
	Significance	***	**	***	**	
C16:0	CO	19.09 ± 0.02^Db^	13.80 ± 0.14^ABa^	12.37 ± 2.28^Aa^	12.37 ± 4.08^Aa^	***
	LB	11.14 ± 0.64^Aa^	12.37 ± 4.18^Aab^	17.38 ± 1.80^Cc^	16.32 ± 2.14^ABbc^	*
	LS	17.52 ± 0.27^Ca^	16.91 ± 0.78^Ba^	16.49 ± 1.60^BCa^	15.17 ± 2.29^ABa^	n.s.
	LSS	13.49 ± 0.16^Ba^	15.65 ± 0.88^ABb^	13.90 ± 0.13^ABa^	19.60 ± 0.24^Bc^	***
	Significance	***	*	*	*	
C17:0	CO	10.94 ± 0.32^Aa^	40.55 ± 0.02^Cc^	59.28 ± 0.33^Bd^	38.50 ± 0.31^Ab^	***
	LB	16.11 ± 0.09^Ba^	41.46 ± 0.21^Cb^	60.54 ± 0.16^Bd^	51.39 ± 0.39^Bc^	***
	LS	29.16 ± 0.05^Ca^	56.94 ± 0.33^Dd^	51.10 ± 0.98^Ab^	54.44 ± 1.07^Cc^	**
	LSS	26.27 ± 0.07^Ca^	35.66 ± 0.04^Ab^	50.43 ± 0.90^Ad^	38.75 ± 0.15^Ac^	***
	Significance	***	***	**	**	
C18:0	CO	149.43 ± 0.04^Aab^	158.89 ± 0.05^Bc^	144.99 ± 0.10^Ba^	149.70 ± 0.36^Cb^	**
	LB	155.99 ± 0.97^Bc^	153.01 ± 0.68^Ab^	153.39 ± 0.45^Cb^	145.89 ± 1.12^Ba^	**
	LS	153.42 ± 2.57^Ba^	151.11 ± 0.23^Aa^	124.95 ± 2.49^Ab^	133.42 ± 2.57^Ac^	***
	LSS	162.73 ± 0.54^Cc^	149.02 ± 6.05^Aa^	158.12 ± 0.00^Dbc^	152.73 ± 0.88^Cab^	
	Significance	**	*	**	**	
C20:0	CO	1.58 ± 0.12^Aa^	5.64 ± 0.40^Bb^	8.37 ± 0.18^Bc^	6.04 ± 0.41^Ab^	***
	LB	2.20 ± 0.09^ABa^	5.91 ± 0.30^Bb^	7.95 ± 0.04^Bc^	8.66 ± 0.39^Bd^	**
	LS	2.72 ± 0.69^Ba^	7.47 ± 0.02^Cb^	6.95 ± 0.16^Ab^	14.05 ± 1.07^Cc^	***
	LSS	3.58 ± 0.01^Ca^	4.91 ± 0.13^Aa^	7.92 ± 0.96^Bb^	15.10 ± 1.02^Cc^	***
	Significance	*	*	*	**	
C14:1	CO	1.53 ± 0.06^Ba^	1.53 ± 0.03^Aa^	3.77 ± 0.00^Ac^	2.33 ± 0.02^Ab^	***
	LB	1.25 ± 0.15^Aa^	2.32 ± 0.01^Bb^	5.57 ± 0.14^Bd^	3.84 ± 0.28^Bc^	***
	LS	2.42 ± 0.01 ^Da^	2.61 ± 0.03^Ca^	5.25 ± 0.35^Bb^	5.06 ± 0.07^Db^	***
	LSS	2.14 ± 0.04^Ca^	2.65 ± 0.14^Cb^	4.04 ± 0.07^Ac^	4.30 ± 0.26^Cc^	**
	Significance	**	**	***	*	
C16:1	CO	18.39 ± 0.32 ^Da^	33.79 ± 0.08^Ab^	53.28 ± 0.62^Bc^	34.04 ± 0.10^Ab^	***
	LB	15.55 ± 0.10^Aa^	36.01 ± 0.66^Bb^	71.55 ± 0.10^Cd^	52.62 ± 0.57^Cc^	***
	LS	17.87 ± 0.04^Ca^	56.88 ± 0.24^Cc^	54.03 ± 0.58^Bb^	60.49 ± 1.43^Dd^	**
	LSS	16.88 ± 0.30^Ba^	33.41 ± 0.07^Ab^	50.53 ± 0.51^Ac^	49.69 ± 1.06^Bc^	***
	Significance	*	***	***	***	
C17:1	CO	14.41 ± 0.03^Aa^	20.11 ± 0.01^Bc^	29.56 ± 0.21^Bd^	19.24 ± 0.08^Ab^	***
	LB	18.73 ± 0.06 ^Da^	20.94 ± 0.11^Cb^	38.42 ± 0.39^Dd^	28.35 ± 0.67^Cc^	***
	LS	16.64 ± 0.02^Ba^	31.55 ± 0.26^Dc^	30.47 ± 0.28^Cb^	31.40 ± 0.17^Dc^	***
	LSS	15.62 ± 0.19^Ca^	19.18 ± 0.02^Ab^	27.72 ± 0.16^Ad^	24.38 ± 0.12^Bc^	***
	Significance	***	***	**	***	
C18:1	CO	254.31 ± 0.64^Ba^	321.96 ± 0.06^Cb^	317.04 ± 5.75^Ab^	384.56 ± 6.54^Bc^	***
	LB	242.20 ± 2.26^Aa^	310.18 ± 1.12^Bb^	313.93 ± 7.66^Ab^	239.65 ± 7.95^Aa^	***
	LS	240.80 ± 2.72^Aa^	321.60 ± 6.07^Cb^	350.47 ± 2.44^Bc^	269.17 ± 8.60^Aa^	**
	LSS	241.18 ± 1.12^Aa^	300.21 ± 4.43^Ab^	324.36 ± 6.31^Ac^	326.93 ± 7.46^Cc^	**
	Significance	***	**	***	***	
C20:1	CO	3.68 ± 0.03^Ca^	6.12 ± 0.12^Bb^	9.47 ± 0.16^Cd^	6.54 ± 0.02^Cc^	***
	LB	3.23 ± 0.09^Aa^	5.93 ± 0.00^Bb^	8.35 ± 0.09^Bd^	7.27 ± 0.06^Dc^	***
	LS	3.74 ± 0.13^Ca^	7.73 ± 0.31^Cc^	7.49 ± 0.27^Ac^	4.97 ± 0.24^Bb^	***
	LSS	3.44 ± 0.13^Ba^	5.02 ± 0.08^Ab^	7.47 ± 0.65^Ac^	3.36 ± 0.09^Aa^	***
	Significance	*	*	*	***	
C18:2n6	CO	43.63 ± 0.51^Ac^	24.28 ± 0.06^Ab^	8.08 ± 0.65^Aa^	7.74 ± 0.94^Aa^	***
	LB	52.29 ± 0.63^Cb^	56.56 ± 0.36^Cc^	12.36 ± 0.06^Ba^	11.97 ± 0.70^Ba^	***
	LS	51.17 ± 0.11^Dc^	55.40 ± 0.21^Dd^	11.53 ± 0.30^Ba^	21.59 ± 3.79^Cb^	***
	LSS	48.25 ± 0.16^Bc^	62.56 ± 0.14^Bd^	18.34 ± 3.30^Cb^	7.60 ± 0.45^Aa^	***
	Significance	***	***	*	**	
C18:2n6T	CO	5.88 ± 1.81^Bb^	2.11 ± 0.09^Ba^	8.18 ± 0.12^Ab^	12.86 ± 0.12^Ac^	**
	LB	8.44 ± 0.18^Cb^	2.09 ± 0.02^Ba^	9.99 ± 0.01^Bc^	11.49 ± 0.79^Ad^	**
	LS	1.66 ± 0.03^Aa^	2.90 ± 0.07^Ca^	9.03 ± 0.20^ABb^	19.22 ± 2.64^Bc^	**
	LSS	1.47 ± 0.02^Aa^	1.93 ± 0.01^Aa^	8.87 ± 0.67^Ab^	22.72 ± 1.58^Bc^	*
	Significance	*	*	*	**	
C18:3n3(ALA)	CO	16.05 ± 002^Ca^	23.27 ± 0.01^Cb^	33.11 ± 0.05^Bd^	27.73 ± 0.03^Bc^	***
	LB	10.05 ± 0.05^Aa^	21.12 ± 0.05^Bb^	35.64 ± 0.22^Cd^	24.11 ± 0.48^Ac^	***
	LS	17.69 ± 0.09 ^Da^	29.88 ± 0.10^Db^	35.45 ± 0.82^Cc^	29.10 ± 0.59^Cb^	***
	LSS	14.12 ± 0.05^Ba^	19.01 ± 0.08^Ab^	30.83 ± 0.72^Ac^	37.05 ± 0.27^Dd^	***
	Significance	***	***	**	**	
C18:3n3(GLA)	CO	0.88 ± 0.11^Aa^	2.86 ± 0.21^Bb^	5.86 ± 0.01^Dd^	4.63 ± 0.07^Cc^	***
	LB	1.18 ± 0.03^Ba^	2.75 ± 0.01^Bb^	4.52 ± 0.05^Cc^	4.73 ± 0.12^Cd^	**
	LS	1.43 ± 0.01^Ca^	3.04 ± 0.03^Cb^	4.01 ± 0.11^Bd^	3.30 ± 0.05^Bc^	**
	LSS	1.14 ± 0.04^Ba^	1.60 ± 0.08^Ab^	2.71 ± 0.27^Ad^	2.26 ± 0.06^Ac^	**
	Significance	**	**	**	***	
C20:2	CO	12.89 ± 0.00 ^Da^	12.82 ± 0.01^Ba^	20.16 ± 0.06^Bb^	25.04 ± 0.38^Ac^	***
	LB	7.82 ± 0.06^Aa^	9.00 ± 0.02^Ab^	18.27 ± 0.27^Ac^	27.82 ± 0.10^Bd^	***
	LS	10.21 ± 0.02^Ba^	14.11 ± 0.05^Db^	39.06 ± 0.88^Dd^	37.50 ± 0.70^Cc^	***
	LSS	11.71 ± 0.03^Ca^	13.73 ± 0.03^Cb^	29.64 ± 0.50^Cc^	45.41 ± 0.39^Dd^	***
	Significance	***	***	**	***	
C20:4n6	CO	0.38 ± 0.04^Aa^	1.56 ± 0.02^Cb^	2.35 ± 0.01^Dd^	1.72 ± 0.05^Ac^	**
	LB	0.49 ± 0.07^Ba^	1.31 ± 0.00^Bb^	1.76 ± 0.02^Cc^	2.29 ± 0.04^Bd^	**
	LS	0.79 ± 0.01^Ca^	1.54 ± 0.08^Cb^	1.70 ± 0.04^Bb^	1.69 ± 0.21^Ab^	***
	LSS	0.45 ± 0.01^Aa^	1.04 ± 0.10^Ab^	1.38 ± 0.03^Ac^	1.88 ± 0.02^Ad^	***
	Significance	*	**	*	*	
C20:5n3	CO	4.14 ± 0.04^Ba^	5.68 ± 0.22^Ab^	10.99 ± 0.01^Ad^	10.17 ± 0.02^Ac^	***
	LB	3.38 ± 0.01^Aa^	5.96 ± 0.12^Bb^	11.74 ± 0.20^Bc^	12.40 ± 0.23^Bd^	**
	LS	5.31 ± 0.02 ^Da^	6.07 ± 0.07^Bb^	12.38 ± 0.19^Cc^	13.58 ± 0.60^Cd^	**
	LSS	5.07 ± 0.13^Ca^	7.23 ± 0.09^Cb^	12.13 ± 0.14^Cc^	12.86 ± 0.48^Bd^	**
	Significance	***	*	*	*	
SFA	CO	224.63 ± 4.18^Aa^	298.12 ± 3.25^Dc^	345.61 ± 2.36^Bd^	284.26 ± 1.20^Ab^	***
	LB	220.46 ± 2.48^Aa^	290.79 ± 3.60^Cb^	367.07 ± 4.02^Cd^	337.63 ± 5.00^Dc^	***
	LS	261.83 ± 4.32^Ba^	265.91 ± 2.20^Aa^	300.73 ± 5.02^Ab^	327.91 ± 1.29^Cc^	***
	LSS	259.89 ± 3.29^Ba^	276.09 ± 4.28^Ba^	331.24 ± 1.87^Bb^	306.85 ± 2.76^Bb^	**
	Significance	***	**	**	**	
MUFA	CO	291.32 ± 2.24^Ba^	383.51 ± 2.20^Cb^	413.12 ± 1.08^Ac^	446.61 ± 3.58^Cd^	***
	LB	280.96 ± 3.08^Aa^	375.38 ± 4.73^Bc^	437.82 ± 2.15^Bd^	331.73 ± 0.77^Ab^	***
	LS	281.47 ± 2.08^Aa^	420.37 ± 4.56^Dc^	447.71 ± 5.87^Cd^	336.12 ± 2.34^Ab^	***
	LSS	279.26 ± 3.06^Aa^	360.47 ± 2.12^Ab^	414.12 ± 1.56^Ad^	408.66 ± 2.24^Bc^	***
	Significance	*	***	**	***	
PUFA	CO	83.35 ± 0.72^Ab^	72.48 ± 1.56^Aa^	88.73 ± 1.88^Ac^	89.89 ± 0.69^Ac^	**
	LB	83.65 ± 1.23^Aa^	98.79 ± 1.45^Cc^	94.28 ± 1.64^Ab^	94.81 ± 1.02^Ab^	***
	LS	88.26 ± 0.88^Ba^	112.94 ± 1.33^Bb^	113.16 ± 1.20^Cb^	113.16 ± 2.60^Bb^	***
	LSS	82.29 ± 0.65^Aa^	107.10 ± 1.24^Bb^	103.19 ± 1.40^Bb^	129.78 ± 2.20^Cc^	***
	Significance	*	***	***	***	
Total FFA	CO	599.30 ± 7.23^Aa^	754.11 ± 4.78^Ab^	847.46 ± 7.38^Ac^	820.76 ± 7.24^Bc^	***
	LB	585.07 ± 5.45^Aa^	764.96 ± 5.04^Ab^	899.17 ± 7.50^Bc^	764.17 ± 7.33^Ab^	***
	LS	631.56 ± 6.12^Ba^	794.22 ± 8.23^Bb^	861.60 ± 6.35^Ac^	777.19 ± 6.49^Ab^	***
	LSS	621.44 ± 5.86^Ba^	743.66 ± 7.47^Ab^	848.55 ± 5.65^Ac^	845.29 ± 6.36^Cc^	***
	Significance	*	***	***	**	

Notes

MUFA, monounsaturated fatty acids; PUFA, polyunsaturated fatty acids; SFA, saturated fatty acid; Total FFA, total free fatty acid content.

Batches: CO: without commercial starter cultures; LB: with single Lactobacillus starter culture (L. sake); LS: with compound starter cultures (*L. sake* + *S. xylosus*); LSS: with compound starter cultures (*L. sake* + *S. xylosus* + *S. carnosus*).

^a,b,c,d^Mean values in the same row (corresponding to the same days of ripening) not followed by a common number differ significantly (*p *<* *0.05).

^A,B,C,D^Mean values in the same row (corresponding to the same batch) not followed by a common letter differ significantly (*p *<* *0.05).

Significance: n.s.: not significant; *(*p *<* *0.05); **(*p *<* *0.01); ***(*p *<* *0.001).

Alpha‐linolenic acid (C18:3 n3) and docosahexaenoic acid (C22:6 n3) can only be acquired through diet and reduce the incidence of diseases, such as diabetes, cancer, and cardiovascular disease (Akpinar, Görgün, & Akpinar, 2009; Jakobsen et al., 2009; Ruiz‐Núñez, Janneke Dijck‐Brouwer, & Muskiet Frits, 2016). For the free fatty acid composition, our results agreed with those reported by Gómez and Lorenzo (2013) that the MUFA were liberated in higher proportions than SFA and PUFA, indicating that the liberation also originates from the triglycerides rich in MUFA. In addition, Marco et al. (2006) observed that higher specific fatty acids were released from the polar fraction, while the majority of FFA derived from the triglycerides, which could be due to the neutral lipids, was the most abundant lipid fraction in intramuscular and subcutaneous fat. However, some of the fatty acids were significantly (*p *<* *0.05) influenced by the use of the starter cultures. During ripening, the MUFA content of the inoculated batches decreased sharply and was significant (*p *<* *0.001) lower than that of the CO, which may be due to β‐oxidation or decomposition into aldehydes and other flavor substances. At the end of ripening, PUFA and total FFA of LSS were significantly higher than those of LB, LS, and CO. In addition, the total FFA of the CO (820.76 mg/100 g of fat) was significantly higher than those of the LB and LS batches (between 764.17 and 777.19 mg/100 g of fat). This suggested that endogenous enzymes played a main role in lipid decomposition, while microbial enzymes could play a promoting role in the composition of free fatty acids.

### 3.5 Sensory analysis

The sensory attributes evaluated by the trained panel are shown in Table 5. The inoculated sausages had significantly higher scores than the CO in all attributes analyzed. And, mixed starter‐inoculated samples were perceived as having better flavor, texture, and overall quality than the LB and control sausages. Table 5 shows the sensory profile of the sausages as affected by the mixed starters of *L. sakei *+ *S. xylosus* and *L. sakei* +* S. xylosus* +* S. carnosus*.

**Table 5:** Effect of different mixed starter cultures on sensory quality of dry fermented mutton sausage

Factor	Appearance	Color	Flavor	Texture
Processing method				
CO	6.50 ± 0.25^a^	6.66 ± 0.20^a^	6.21 ± 0.42^a^	6.56 ± 0.32^a^
LB	7.42 ± 0.24^b^	7.35 ± 0.15^b^	7.12 ± 0.31^b^	7.23 ± 0.41^ab^
LS	7.62 ± 0.12^b^	7.65 ± 0.21^b^	7.78 ± 0.24^c^	7.45 ± 0.50^b^
LSS	7.65 ± 0.45^b^	7.69 ± 0.27^b^	7.86 ± 0.26^c^	7.45 ± 0.20^b^

Notes

Batches: CO: without commercial starter cultures; LB: with single Lactobacillus starter culture (*L. sake*); LS: with compound starter cultures (*L. sake* + *S. xylosus*); LSS: with compound starter cultures (*L. sake* + *S. xylosus* + *S. carnosus*).

^a,b,c^Mean values in the same row (corresponding to the same days of ripening) not followed by a common number differ significantly (*p *<* *0.05).

## 4 CONCLUSIONS

The LAB and staphylococci counts were higher in the inoculated batches and were the most dominant bacteria. Adding starter cultures, especially the mixed starter cultures (*L*. *sakei *+ *S. xylosus* and *L. sakei* +* S. xylosus* +* S. carnosus*), accelerated acidification and decrease in water activity and reduced lipid oxidation of dry fermented mutton sausages, which inhibited the growth of Enterobacteriaceae and improved hygienic quality. The mixed starter cultures, especially *L. sakei *+ *S. xylosus *+ *S. carnosus*, promoted proteolysis and improved the content of total free amino acids, total free fatty acids, and polyunsaturated fatty acids. These results suggest that the mixed starter cultures (*L. sakei* + *S*. *xylosus* and *L. sakei* +* S. xylosus* +* S. carnosus*) were conducive to improving the quality and safety of dry fermented mutton sausages.

## CONFLICTS OF INTEREST

The authors declare no conflict of interest or relationship, financial or otherwise.

## ETHICAL APPROVAL

It is not applicable.
